# Autonomic Dysfunction as a Potential Pathophysiological Link Between Chronic Low Back Pain and Coronary Artery Disease: A Systematic Review

**DOI:** 10.7759/cureus.110661

**Published:** 2026-06-11

**Authors:** Waqas Alauddin, Rahul Saxena, Arushi Saxena, Sayali Khairnar, Srisaisantoshini Sankaranarayanan, Brishabh R Prajesh, Fahad Shaikh, Soumya Singh

**Affiliations:** 1 Physiology, Dr. N. Y. Tasgaonkar Institute of Medical Science, Karjat, IND; 2 Physiology, Varun Arjun Medical College and Rohilkhand Hospital, Shahjahanpur, IND; 3 Anesthesiology and Critical Care, Varun Arjun Medical College and Rohilkhand Hospital, Shahjahanpur, IND; 4 Musculoskeletal Physiotherapy, Dr. N. Y. Tasgaonkar College of Physiotherapy, Karjat, IND; 5 Cardiovascular and Pulmonary Physiotherapy, Dr. N. Y. Tasgaonkar College of Physiotherapy, Karjat, IND; 6 Medicine, Naraina Medical College and Research Centre, Kanpur, IND; 7 Medicine, N. K. P. Salve Institute of Medical Sciences and Research Centre, Nagpur, IND; 8 Oral Medicine and Radiology, Institute of Dental Studies and Technologies, Modinagar, IND

**Keywords:** autonomic nervous system (ans), cardiovascular autonomic dysfunction (cad), chronic low back pain (clbp), coronary artery disease (cad), heart rate variability (hrv), risk factors of cardiovascular diseases, sympathetic and parasympathetic activity

## Abstract

Chronic low back pain (CLBP) is a leading cause of disability worldwide and is increasingly recognized as a condition associated with systemic physiological alterations beyond musculoskeletal dysfunction. Emerging evidence suggests that autonomic nervous system dysregulation may be associated with impaired cardiovascular regulation and increased cardiovascular risk in individuals with CLBP. This systematic review aimed to evaluate the current evidence regarding autonomic dysfunction in CLBP and its potential association with cardiovascular disease. A comprehensive literature search was conducted according to the PRISMA 2020 guidelines across PubMed/MEDLINE, Scopus, Web of Science, EMBASE, CENTRAL, CINAHL, EBSCOhost, and Google Scholar from database inception through January 2026, along with gray literature sources. Studies evaluating autonomic or cardiovascular autonomic function in adults with CLBP were included. A total of 10 studies met the inclusion criteria. Most studies demonstrated reduced heart rate variability, diminished vagal activity, sympathetic predominance, impaired autonomic regulation, and delayed cardiovascular recovery in individuals with CLBP compared with healthy controls. Several studies also reported associations between autonomic dysfunction and pain-related disability, catastrophizing, kinesiophobia, and altered pain modulation. Population-based evidence suggested increased prevalence of coronary heart disease and myocardial infarction among individuals with CLBP, while interventional studies demonstrated improvements in autonomic regulation following yoga-based rehabilitation and spinal manipulative therapy. Overall, the certainty of evidence ranged from low to moderate because of methodological heterogeneity and the predominance of observational study designs. Current evidence suggests that CLBP is associated with clinically relevant autonomic and cardiovascular alterations. These findings highlight the importance of incorporating autonomic and cardiovascular assessment into multidisciplinary management strategies while emphasizing the need for further prospective and mechanistic research.

## Introduction and background

Chronic low back pain (CLBP) is among the most prevalent musculoskeletal disorders worldwide and remains a leading cause of disability, functional limitation, reduced quality of life, and healthcare utilization across both developed and developing nations [1]. Beyond its substantial socioeconomic burden, CLBP is increasingly recognized as a complex biopsychosocial condition involving not only structural and mechanical abnormalities but also neurophysiological, psychological, and systemic alterations [2]. Persistent nociceptive input, central sensitization, maladaptive pain processing, emotional stress, sleep disturbances, and physical deconditioning are believed to contribute to the chronicity and multidimensional nature of CLBP [3].

In recent years, growing attention has been directed toward the role of the autonomic nervous system (ANS) in chronic pain disorders. The ANS plays a fundamental role in maintaining cardiovascular homeostasis through coordinated sympathetic and parasympathetic regulation of heart rate, vascular tone, blood pressure, and physiological stress responses [4]. Noninvasive measures such as heart rate variability (HRV), baroreflex sensitivity, blood pressure variability, and heart rate recovery (HRR) are widely used to evaluate autonomic function and cardiovascular adaptability [5]. Reduced HRV and impaired vagal modulation have been consistently associated with increased cardiovascular morbidity, coronary artery disease, myocardial infarction, arrhythmias, and all-cause mortality across multiple chronic disease states [6].

Emerging evidence suggests that individuals with CLBP frequently exhibit altered autonomic regulation characterized by sympathetic predominance and diminished parasympathetic activity [7-10]. Gockel et al. demonstrated that patients with greater disability related to CLBP had significantly lower HRV and reduced parasympathetic activity, indicating impaired autonomic balance associated with chronic pain-related functional impairment [7]. Similarly, several observational and comparative studies have reported reduced vagal modulation, elevated sympathetic activity, delayed HRR, and impaired cardiovascular autonomic regulation in individuals with CLBP compared with healthy controls [8-11]. Furthermore, psychosocial factors, including pain catastrophizing, fear-avoidance behavior, disability severity, anxiety, and impaired descending nociceptive inhibitory mechanisms, may further contribute to autonomic dysregulation in chronic pain populations [9,10].

Importantly, chronic autonomic imbalance may have broader cardiovascular implications. Sustained sympathetic activation and reduced parasympathetic tone have been linked to endothelial dysfunction, increased myocardial oxygen demand, systemic inflammation, impaired cardiovascular recovery, and accelerated atherosclerotic progression [12]. Fernandez et al., in a large co-twin control study, reported a significantly increased prevalence of coronary heart disease and myocardial infarction among individuals with CLBP, even after adjustment for shared genetic and familial factors [13]. These findings suggest that autonomic dysfunction may represent a potential pathophysiological link between chronic pain and cardiovascular disease.

Although numerous studies have investigated autonomic dysfunction in CLBP, the available evidence remains heterogeneous and fragmented, with substantial variability in study design, autonomic assessment techniques, participant characteristics, and cardiovascular outcome measures. To the best of our knowledge, no comprehensive synthesis has specifically focused on the relationship between autonomic dysregulation in CLBP and its potential cardiovascular implications. Therefore, the present systematic review aimed to critically evaluate and synthesize the current evidence regarding autonomic dysfunction in CLBP and its potential association with cardiovascular disease and impaired cardiovascular regulation.

## Review

Methods

This systematic review was conducted in accordance with the PRISMA 2020 guidelines and adhered to the PRISMA-P recommendations for systematic review protocol development [14]. The research question was formulated using the population, intervention/exposure, comparison, and outcome (PICO) framework, focusing on the association between autonomic dysfunction in individuals with CLBP and its potential cardiovascular implications.

Literature Search Strategy

A comprehensive and systematic literature search was performed across multiple electronic databases, including PubMed/MEDLINE, Scopus, Web of Science, EBSCO Host, Cochrane Central Register of Controlled Trials (CENTRAL), CINAHL, EMBASE, and Google Scholar. Studies published from database inception through January 2026 were considered eligible for inclusion.

The search strategy incorporated combinations of Medical Subject Headings (MeSH), free-text keywords, and Boolean operators (“AND” and “OR”) to maximize sensitivity and comprehensiveness. Search terms included “chronic low back pain”, “persistent low back pain”, “autonomic dysfunction”, “autonomic nervous system”, “heart rate variability”, “sympathetic activity”, “parasympathetic activity”, “cardiovascular function”, “cardiovascular risk”, and “baroreflex sensitivity”. Synonymous terms were combined using “OR”, while distinct concepts were linked using “AND”. Detailed search strategies for individual databases are provided in Appendix A.

To minimize publication bias, gray literature sources, including OpenGrey, WorldCat, ClinicalTrials.gov, the WHO International Clinical Trials Registry Platform (ICTRP), PROSPERO, the Open Science Framework (OSF) Registries, and the ISRCTN Registry, were additionally searched. Manual reference screening and forward citation tracking of relevant studies were also conducted to identify potentially eligible articles [15-17].

Eligibility Criteria

Inclusion and exclusion criteria: Studies were considered eligible for inclusion if they were original research articles published between 2000 and January 2026 involving adult participants aged 18 years or older diagnosed with CLBP lasting longer than 12 weeks. Included studies were required to evaluate autonomic or cardiovascular autonomic function using validated assessment methods such as HRV, baroreflex sensitivity, blood pressure variability, resting heart rate, sympathetic skin response, or related autonomic measures. Observational, cross-sectional, cohort, case-control, longitudinal, and clinical trial designs were considered eligible. Studies comparing individuals with CLBP to healthy controls or investigating cardiovascular implications associated with autonomic dysfunction were also included. Only studies published in the English language were considered for review.

Studies were excluded if they involved acute or subacute low back pain populations, postoperative low back pain, spinal cord injury, inflammatory or malignant spinal disorders, or neurological conditions unrelated to CLBP. Case reports, conference abstracts without accessible full texts, editorials, commentaries, narrative reviews, systematic reviews, and animal studies were also excluded. Additionally, studies that did not report autonomic or cardiovascular outcome measures or lacked sufficient methodological or outcome-related data for evaluation were excluded from the review.

Study Selection

Two independent reviewers screened titles and abstracts for eligibility. Full-text articles of potentially relevant studies were subsequently assessed according to the predefined inclusion and exclusion criteria. Any disagreements between reviewers were resolved through discussion and consensus, with consultation from a third reviewer when required.

The study selection process is summarized in the PRISMA flow diagram. The initial literature search identified 4,050 records from electronic databases and 117 additional records from gray literature sources and trial registries. Following removal of duplicate records and screening exclusions, 10 studies met the eligibility criteria and were included in the final qualitative synthesis.

Data Extraction

Data extraction was independently performed by two reviewers using a standardized and predefined extraction form. Extracted variables included author information, publication year, country of origin, study design, sample characteristics, duration and severity of CLBP, comparison groups, autonomic assessment methods, cardiovascular outcome measures, and principal findings.

Autonomic variables extracted included HRV parameters (e.g., root mean square of successive differences (RMSSD), standard deviation of NN intervals (SDNN), low frequency (LF)/high frequency (HF) ratio, HF power), baroreflex sensitivity, resting heart rate, sympathetic and parasympathetic activity indices, and autonomic reflex measures. Cardiovascular outcomes included blood pressure responses, myocardial workload, HRR, vascular function, and indicators of cardiovascular risk. Any discrepancies during extraction were resolved through consensus.

Quality Assessment and Risk of Bias

Methodological quality and risk of bias of the included observational studies were evaluated using the Newcastle-Ottawa Scale (NOS), which assesses study quality across three domains: participant selection, comparability, and outcome/exposure assessment [18]. Two reviewers independently conducted the quality assessment, and disagreements were resolved by consensus. Studies were categorized as moderate or high methodological quality based on NOS scoring criteria.

Certainty of Evidence

The certainty of evidence for major outcomes was assessed using the Grading of Recommendations Assessment, Development and Evaluation (GRADE) framework [19]. Evidence quality was evaluated across the domains of risk of bias, inconsistency, indirectness, imprecision, and publication bias and categorized as high, moderate, low, or very low certainty.

Data Synthesis

Due to substantial heterogeneity in study designs, participant characteristics, autonomic assessment techniques, cardiovascular outcome measures, and statistical methodologies, quantitative meta-analysis was not considered appropriate. Therefore, a qualitative narrative synthesis was undertaken.

Findings were organized into thematic domains, including autonomic dysfunction in CLBP, alterations in sympathetic and parasympathetic activity, HRV and cardiovascular autonomic regulation, psychosocial influences on autonomic function, cardiovascular risk implications, and effects of therapeutic interventions. Similarities, inconsistencies, and emerging mechanistic patterns across studies were systematically synthesized to provide an integrated understanding of autonomic and cardiovascular dysregulation in CLBP populations.

Ethical Considerations and Registration

As this study analyzed previously published data, ethical approval and informed consent were not required. The review protocol was prospectively registered with the International Prospective Register of Systematic Reviews (PROSPERO) under registration number CRD420261398092 to enhance methodological transparency and reproducibility and reduce reporting bias.

Results

Study Identification

**Figure 1:**
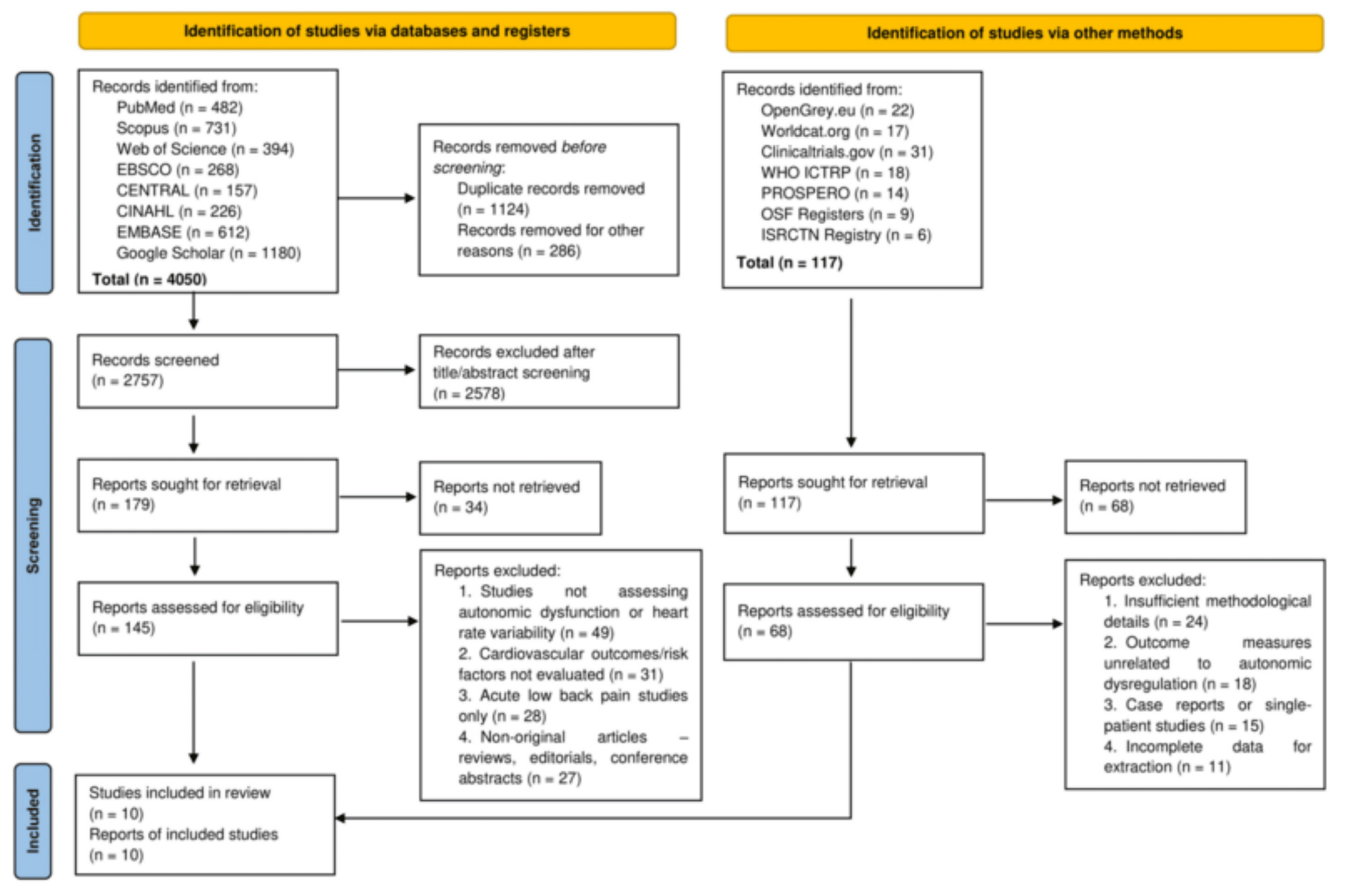
PRISMA flow diagram of study selection process

The search strategy identified a total of 4050 records from electronic databases and 117 records from additional sources. After removal of duplicates (n = 1124) and other exclusions (n = 286), 2757 records were retained for screening. Following title and abstract screening, 2578 records were excluded. A total of 179 reports were retrieved for further evaluation, among which 34 reports could not be accessed. Consequently, 145 full-text articles underwent eligibility assessment. Among these, 135 studies were excluded because autonomic dysfunction or HRV was not assessed (n = 49), cardiovascular outcomes or risk factors were not evaluated (n = 31), studies focused only on acute low back pain (n = 28), or the articles were non-original publications such as reviews, editorials, or conference abstracts (n = 27). An additional 3276 full-text articles were identified through gray literature searches; however, none satisfied the inclusion criteria. Ultimately, 10 studies were included in the qualitative synthesis (Figure 1).

Characteristics of Included Studies

The review included 10 studies published between 2008 and 2026 investigating neurophysiological alterations and cardiovascular implications in individuals with CLBP [7-11,13,20-23]. The included studies primarily consisted of observational, cross-sectional, comparative, and interventional designs. Sample sizes ranged from small clinical cohorts to large population-based twin registries. Most studies included adults with CLBP lasting longer than three months and compared findings with healthy controls or evaluated cardiovascular associations within CLBP populations.

HRV was the most frequently utilized physiological assessment method across studies [7-11,20-23]. Additional cardiovascular and regulatory measures included baroreflex sensitivity, blood pressure variability, HRR, rate-pressure product (RPP), myocardial workload, and sympathovagal indices [9,12,16]. Several studies demonstrated diminished vagal activity, sympathetic overactivity, impaired cardiovascular recovery, and altered physiological regulation among individuals with CLBP [7,8,10,11,22]. Psychosocial variables such as disability severity, catastrophizing, and kinesiophobia were also associated with impaired physiological control [7,9,21].

Results of Search

The PRISMA-guided selection process resulted in 10 studies being included in the final analysis (Figure 1). These studies encompassed observational and mechanistic designs examining autonomic dysregulation, HRV, and cardiovascular risk in individuals with CLBP [7-11,13,20-23].

Methodological Quality and Risk of Bias

The methodological quality of included studies was assessed using the NOS (Table 1). Overall, methodological quality ranged from moderate to high, with NOS scores ranging from 5 to 9 out of a maximum possible score of 9 [7-11,13,20-23]. Higher-quality studies demonstrated clearly defined CLBP populations, validated autonomic assessment methods, appropriate comparison groups, and adjustment for major confounding variables, including age, sex, smoking status, body mass index, and physical activity [8,9,13].

**Table 1:** NOS quality assessment of included studies Quality assessment of the included studies using the NOS. Higher scores indicate better methodological quality across selection, comparability, and outcome domains. NOS, Newcastle-Ottawa Scale

Study	Selection (0-4)	Comparability (0-2)	Outcome/exposure (0-3)	Total	Quality
Gockel et al. [7]	3	1	2	6	Moderate
Fernández-Morales et al. [8]	3	2	2	7	Moderate to high
Pontes-Silva et al. [9]	4	2	2	8	High
Martins et al. [10]	2	1	2	5	Moderate
Espejo-Antúnez et al. [11]	3	1	2	6	Moderate
Fernandez et al. [13]	4	2	3	9	High
Telles et al. [20]	3	1	2	6	Moderate
Younes et al. [21]	3	2	2	7	Moderate to high
Rodrigues et al. [22]	2	1	2	5	Moderate
Espejo-Antúnez et al. [23]	3	1	2	6	Moderate

The study by Fernandez et al. (2016) demonstrated the highest methodological quality owing to its large population-based co-twin design and extensive adjustment for genetic and environmental confounders [13]. Common methodological limitations included cross-sectional study designs, small sample sizes, inadequate adjustment for psychosocial confounders, and limited longitudinal follow-up [7,10,11,22,23]. Despite these limitations, most studies employed standardized autonomic and cardiovascular assessment techniques, supporting the reliability of the overall findings.

Certainty of Evidence

The certainty of evidence was evaluated using the GRADE framework and was generally categorized as moderate across most outcomes (Table 2). Evidence supporting autonomic dysfunction in CLBP populations was relatively consistent, particularly regarding reduced HRV, sympathetic predominance, and diminished parasympathetic activity [7,10,11,15,20,22].

**Table 2:** Certainty of evidence assessment summary Certainty of evidence was assessed using the GRADE framework across major autonomic and cardiovascular outcomes. Certainty levels reflect methodological quality, consistency of findings, risk of bias, and strength of available evidence. CLBP, chronic low back pain; GRADE, Grading of Recommendations Assessment, Development and Evaluation; HRV, heart rate variability

Outcome	Evidence type	Overall certainty	Key findings	References
Reduced HRV in CLBP	Observational cross-sectional studies	Moderate	Individuals with CLBP demonstrated lower HRV and reduced parasympathetic activity	[7]
Sympathetic predominance and reduced vagal activity	Primarily observational evidence	Moderate to high	Most studies reported autonomic imbalance with increased sympathetic activity and reduced vagal modulation	[7,20]
Association between CLBP and cardiovascular disease	Population-based co-twin study	High	Increased prevalence of myocardial infarction and coronary heart disease observed in CLBP populations	[13]
Cardiovascular autonomic dysregulation	Observational and cross-sectional studies	Moderate	Impaired cardiovascular autonomic control, abnormal blood pressure variability, and altered hemodynamic responses reported	[10,21,22]
Disability severity and autonomic dysfunction	Cross-sectional clinical evidence	Moderate	Greater pain-related disability was associated with reduced HRV and impaired autonomic regulation	[7,9,11,13]
Potential long-term cardiovascular risk	Indirect observational evidence	Low to moderate	Chronic autonomic dysregulation may be associated with increased cardiovascular morbidity	[8,23]

The strongest evidence was observed for the association between CLBP and cardiovascular disease risk, particularly coronary heart disease and myocardial infarction, supported by large population-based studies with rigorous statistical adjustment [13]. However, certainty was downgraded in several domains due to the predominance of observational and cross-sectional designs, heterogeneity in autonomic assessment techniques, small sample sizes, and limited longitudinal evidence [7-11,21-23]. Overall, the available evidence supports a clinically relevant association between CLBP, autonomic dysregulation, and altered cardiovascular function.

Synthesis of findings

Autonomic Dysfunction in CLBP

All included studies demonstrated evidence of altered ANS activity in individuals with CLBP [7-11,20-23]. The most commonly reported findings included diminished vagal activity, sympathetic overactivity, and impaired physiological regulation. These alterations were reflected by decreased HRV indices such as RMSSD, SDNN, and HF power, along with elevated LF/HF ratios and stress indices [7,8,11,22]. Individuals with greater pain-related disability consistently demonstrated more pronounced physiological impairment, suggesting that chronic pain severity and functional limitation may adversely influence autonomic control.

HRV and Cardiovascular Regulation

HRV emerged as the principal physiological marker across the included studies [7-11,20-23]. Multiple investigations reported significantly lower HRV in individuals with CLBP compared with healthy controls, indicating reduced physiological adaptability and impaired vagal modulation [7,8,11,22]. Several studies additionally identified abnormalities in cardiovascular function, including delayed HRR following exercise, impaired baroreflex sensitivity, increased myocardial workload, abnormal blood pressure responses, and impaired neurocardiovascular control [10,11,20]. These findings suggest that CLBP may adversely influence both resting and exercise-related cardiovascular performance.

Psychosocial Factors and Physiological Dysregulation

Psychological and behavioral variables such as disability severity, pain catastrophizing, fear-avoidance behavior, and kinesiophobia were consistently associated with impaired physiological regulation [7,9,21]. Several studies demonstrated correlations between psychosocial distress and reduced HRV, suggesting a complex interaction between chronic pain, emotional regulation, and neurophysiological activity [9,21]. These observations support the biopsychosocial model of CLBP, in which psychological, neurological, and physiological mechanisms collectively contribute to symptom persistence and functional impairment.

Cardiovascular Implications

Several included studies suggested that individuals with CLBP may have an elevated risk of cardiovascular disease, including coronary heart disease and myocardial infarction [10,11,13]. Persistent sympathetic overactivity, reduced vagal tone, delayed cardiovascular recovery, and altered hemodynamic responses were proposed as potential contributing mechanisms [10,11,13]. Collectively, these findings indicate that CLBP may represent a systemic condition with clinically important cardiovascular implications extending beyond localized musculoskeletal dysfunction.

Effects of Therapeutic Interventions

Interventional studies evaluating spinal manipulative therapy (SMT) and yoga-based rehabilitation demonstrated improvements in HRV parameters and physiological adaptability following treatment [20,23]. Enhanced vagal modulation and improved neurocardiovascular regulation were reported after these interventions [20,23]. These findings suggest that therapeutic strategies targeting physiological restoration may provide additional clinical benefits in individuals with CLBP.

Summary of Key Findings

Overall, the included studies consistently demonstrated that CLBP is associated with clinically relevant disturbances in neurophysiological and cardiovascular regulation [7-11,13,20-23]. Greater pain-related disability, psychosocial distress, catastrophizing, and kinesiophobia were associated with more pronounced physiological impairment, further supporting the multidimensional biopsychosocial nature of CLBP [7,9,21]. Emerging evidence also suggests that these alterations may be linked to impaired cardiovascular recovery and increased cardiovascular risk, including coronary heart disease and myocardial infarction [10,11,13]. Furthermore, interventions such as exercise-based rehabilitation, yoga therapy, and spinal manipulative techniques demonstrated promising improvements in HRV and cardiovascular adaptability [20,23].

The included studies (Table 3) primarily investigated the relationship between CLBP, physiological regulation, and cardiovascular function using observational, cross-sectional, experimental, and interventional study designs. Most studies evaluated HRV parameters such as RMSSD, SDNN, HF, LF, LF/HF ratio, SD1, SD2, and baroreflex sensitivity. Several investigations additionally assessed cardiovascular recovery indices, including HRR, blood pressure responses, myocardial workload, and RPP. Across studies, individuals with CLBP consistently demonstrated impaired vagal modulation, sympathetic overactivity, reduced physiological adaptability, and delayed cardiovascular recovery compared with healthy controls. Collectively, these findings suggest that CLBP is not merely a localized musculoskeletal disorder but a complex systemic condition associated with clinically important neurophysiological and cardiovascular alterations, emphasizing the importance of multidisciplinary rehabilitation strategies targeting both pain management and physiological restoration.

**Table 3:** Summary of included studies on CLBP, autonomic dysfunction, and cardiovascular outcomes CLBP, chronic low back pain; HRR, heart rate recovery; SMT, spinal manipulative therapy

Study	Study design	Main autonomic findings	Cardiovascular findings	Key conclusion
Gockel et al. [7]	Cross-sectional	Reduced HRV and vagal activity; increased sympathetic dominance in patients with higher disability	Altered autonomic cardiovascular balance	Greater CLBP-related disability was associated with autonomic imbalance
Fernández-Morales et al. [8]	Comparative cross-sectional	Altered HRV patterns in CLBP and spinal pain disorders	Disturbed cardiovascular autonomic modulation	Chronic spinal pain conditions demonstrated altered autonomic function
Pontes-Silva et al. [9]	Cross-sectional	Altered HRV and impaired pain modulation mechanisms	Abnormal blood pressure responses	Autonomic dysregulation may contribute to dysfunctional pain inhibition
Martins et al. [10]	Cross-sectional	Impaired autonomic recovery after exercise	Delayed cardiovascular recovery and increased workload	Cardiovascular and autonomic impairment may be important considerations in CLBP rehabilitation
Espejo-Antúnez et al. [11]	Cross-sectional	Reduced HRV and delayed HRR	Increased myocardial workload and impaired cardiovascular recovery	CLBP was associated with impaired cardiovascular autonomic regulation
Fernandez et al. [13]	Co-twin cross-sectional study	Indirect evidence of autonomic dysregulation	Increased prevalence of myocardial infarction and coronary heart disease	CLBP may be associated with increased cardiovascular disease risk
Telles et al. [20]	Randomized sham-controlled trial	Increased vagal modulation after SMT	Improved autonomic cardiovascular regulation	SMT improved parasympathetic activity and sympathovagal balance
Younes et al. [21]	Comparative cross-sectional	HRV abnormalities associated with catastrophizing and kinesiophobia	Altered autonomic regulation	Psychosocial factors were associated with autonomic dysfunction in CLBP
Rodrigues et al. [22]	Observational comparative	Reduced parasympathetic activity and increased sympathetic activity	Impaired autonomic balance	CLBP demonstrated sympathovagal imbalance compared with controls
Espejo-Antúnez et al. [23]	Experimental intervention	Improved HRV and autonomic balance following yoga intervention	Improved cardiovascular autonomic modulation	Yoga-based rehabilitation may improve autonomic regulation

Discussion

This systematic review synthesized current evidence regarding neurophysiological and cardiovascular alterations in individuals with CLBP. The findings consistently demonstrated clinically relevant disturbances in autonomic regulation, including reduced HRV, impaired vagal modulation, sympathetic predominance, and altered cardiovascular autonomic control [7-11,20-23]. Collectively, these findings suggest that CLBP extends beyond a localized musculoskeletal disorder and may involve broader systemic and neurophysiological alterations with potential cardiovascular implications.

Reduced HRV emerged as one of the most consistent findings across the included studies [7,11,12,15,16]. HRV is a well-established noninvasive marker of physiological adaptability and cardiovascular health, reflecting the dynamic interaction between sympathetic and parasympathetic nervous system activity. Lower HRV has previously been associated with increased cardiovascular morbidity, arrhythmias, coronary artery disease, myocardial infarction, and all-cause mortality [5,12,24-27]. Therefore, the reduced HRV observed in individuals with CLBP may indicate impaired physiological resilience and reduced autonomic flexibility, potentially increasing long-term cardiovascular vulnerability.

Several studies also reported enhanced sympathetic drive and diminished vagal modulation among individuals with CLBP [7,8,11,22]. Persistent sympathetic activation has been proposed to influence chronic pain sensitization through mechanisms including increased muscle tension, impaired microcirculation, inflammatory activation, and altered nociceptive processing [28,29]. Simultaneously, reduced vagal influence may impair stress recovery and endogenous anti-inflammatory responses. This disruption in sympathovagal regulation may therefore represent a potential mechanism linking chronic pain with cardiovascular impairment.

The mechanisms underlying these physiological alterations are likely multifactorial. Persistent nociceptive input from lumbar structures may promote central sensitization and sustained activation of stress-responsive neural pathways involving the hypothalamic-pituitary-adrenal axis and central autonomic networks [3,29]. In addition, psychosocial factors such as anxiety, depression, catastrophizing, kinesiophobia, and fear-avoidance behaviors may further aggravate neurophysiological dysregulation [9,21]. Several included studies demonstrated significant associations between disability severity, psychosocial distress, and impaired physiological regulation, supporting the multidimensional biopsychosocial model of chronic pain [7,9,21].

An important finding of this review was the observed relationship between CLBP and cardiovascular disease. Fernández-Morales et al. reported an increased prevalence of coronary heart disease and myocardial infarction among individuals with CLBP, even after adjustment for shared genetic and environmental factors [8]. Similarly, studies evaluating cardiovascular recovery and hemodynamic responses identified delayed HRR, impaired baroreflex sensitivity, increased myocardial workload, and abnormal blood pressure responses in CLBP populations [10,11]. These findings suggest that persistent neurophysiological dysregulation may be associated with impaired cardiovascular adaptability and adverse cardiovascular changes over time.

Several physiological mechanisms may explain these cardiovascular alterations. Sustained sympathetic overactivity may increase resting heart rate, vascular resistance, myocardial oxygen demand, oxidative stress, endothelial dysfunction, and systemic inflammation, all of which are recognized contributors to cardiovascular disease progression [12,29]. Reduced vagal influence may additionally impair cardioprotective and anti-inflammatory pathways. Together, these changes may partly explain the elevated cardiovascular risk observed in chronic pain populations.

Another important observation from this review was the potential reversibility of these alterations through therapeutic interventions. Studies evaluating yoga-based rehabilitation and SMT demonstrated improvements in HRV indices and vagal modulation following treatment [20,23]. These findings suggest that interventions targeting physiological regulation may provide benefits beyond pain reduction alone. Mind-body therapies, exercise-based rehabilitation, breathing exercises, and stress-reduction strategies may positively influence cardiovascular adaptability and neurophysiological regulation in individuals with CLBP.

Despite the overall consistency of findings, variability existed across the included studies regarding the magnitude and pattern of autonomic dysfunction observed in individuals with CLBP. Differences in HRV outcomes may be attributable to methodological heterogeneity, including variations in participant demographics, pain duration and severity, psychosocial status, physical activity levels, medication use, and comorbid cardiovascular risk factors. Additionally, studies utilized different autonomic assessment protocols, HRV parameters, breathing conditions, exercise testing methods, and analytical techniques, which may have influenced the comparability of findings. Some studies evaluated resting autonomic function, whereas others assessed post-exercise cardiovascular recovery or intervention-induced changes, further contributing to heterogeneity. These methodological differences likely explain variations in reported autonomic indices across studies and highlight the need for standardized assessment protocols in future research.

The findings of this review have important clinical implications. Clinicians managing individuals with CLBP should recognize that chronic pain may be associated with broader systemic and cardiovascular consequences. Incorporating cardiovascular and physiological assessment into multidisciplinary pain management strategies may help identify patients at elevated cardiovascular risk. HRV analysis and related physiological markers may also serve as useful adjunctive tools for risk stratification and monitoring treatment response in chronic pain populations.

Future research should prioritize large-scale prospective longitudinal studies investigating temporal relationships between chronic pain, neurophysiological dysregulation, and cardiovascular disease development. Standardization of physiological assessment techniques, inclusion of objective cardiovascular biomarkers, and evaluation of intervention-induced changes may further strengthen the evidence base. Additional mechanistic studies exploring neuroimmune and inflammatory pathways linking CLBP and cardiovascular dysfunction are also warranted.

Overall, this review highlights altered physiological regulation as a potentially important factor associated with CLBP and cardiovascular health. Recognition of these systemic alterations in CLBP may contribute to more comprehensive, multidisciplinary, and prevention-oriented approaches to chronic pain management.

Limitations

Several limitations of this systematic review should be acknowledged. First, most included studies were observational and cross-sectional in design, limiting causal interpretation between CLBP, autonomic dysfunction, and cardiovascular outcomes [7-11,20-23]. Second, substantial methodological heterogeneity existed across studies regarding participant characteristics, CLBP definitions, autonomic assessment methods, cardiovascular outcome measures, and analytical approaches, which precluded quantitative meta-analysis. Additionally, several studies involved relatively small sample sizes and limited longitudinal follow-up, reducing generalizability and restricting evaluation of long-term cardiovascular implications. Adjustment for confounding factors such as anxiety, depression, sleep disturbances, medication use, and lifestyle-related cardiovascular risk factors was also inconsistent across studies. Publication and language bias may have influenced the findings because only English-language studies were included. Despite these limitations, the present review provides a comprehensive synthesis of current evidence regarding autonomic dysfunction and cardiovascular implications in CLBP and highlights important directions for future prospective and mechanistic research.

## Conclusions

This systematic review demonstrates that CLBP is consistently associated with ANS dysfunction, including sympathetic overactivity, diminished vagal tone, reduced HRV, and impaired cardiovascular autonomic regulation. The available evidence suggests that these alterations may be associated with impaired cardiovascular recovery and increased cardiovascular risk in individuals with CLBP. Beyond musculoskeletal impairment, CLBP appears to involve broader neurophysiological and systemic changes influenced by persistent nociceptive stimulation, psychosocial stressors, and maladaptive physiological responses. Several studies also identified associations between autonomic dysfunction and pain-related disability, catastrophizing, kinesiophobia, and altered pain modulation, supporting the multidimensional biopsychosocial nature of CLBP. Interventional studies further suggest that therapies such as yoga-based rehabilitation and SMT may improve autonomic regulation and cardiovascular function. Although current evidence supports an association between CLBP and neurocardiovascular alterations, interpretation remains limited by methodological heterogeneity, observational study designs, and limited longitudinal data. Further prospective and mechanistic studies are required to clarify underlying biological pathways and long-term cardiovascular implications. Overall, these findings suggest that CLBP should not be viewed solely as a localized musculoskeletal disorder but also as a condition with important systemic and cardiovascular implications.

## Appendices

Appendix A: Search strategies

**Table 4:** Detailed search strategies for individual databases The search strategy used combinations of Medical Subject Headings (MeSH), database-specific subject headings, free-text keywords, truncation symbols, and Boolean operators (“AND”, “OR”) to identify studies on autonomic dysfunction, chronic low back pain, and cardiovascular implications. Searches were conducted from January 2000 to May 2026 across electronic databases and gray literature sources.

Database	Search strategy
PubMed/MEDLINE	((“Chronic Low Back Pain”[Mesh] OR “Low Back Pain”[Mesh] OR “chronic low back pain” OR “chronic back pain” OR CLBP OR “persistent low back pain” OR “non-specific low back pain”) AND (“Autonomic Nervous System Diseases”[Mesh] OR “Dysautonomia”[Mesh] OR “autonomic dysfunction” OR dysautonomia OR “autonomic nervous system” OR sympathetic OR parasympathetic OR “heart rate variability” OR HRV OR “baroreflex sensitivity” OR “cardiovascular autonomic control”) AND (“Cardiovascular System”[Mesh] OR “Cardiovascular Diseases”[Mesh] OR cardiovascular OR cardiac OR hemodynamic OR haemodynamic OR “blood pressure variability” OR hypertension OR “resting heart rate” OR “vascular function”)) AND (“2000/01/01”[Date - Publication] : “2026/12/31”[Date - Publication])
Scopus	TITLE-ABS-KEY ((“chronic low back pain” OR “chronic back pain” OR CLBP OR “persistent low back pain” OR “non-specific low back pain”) AND (“autonomic dysfunction” OR dysautonomia OR “autonomic nervous system” OR sympathetic OR parasympathetic OR “heart rate variability” OR HRV OR “baroreflex sensitivity”) AND (cardiovascular OR cardiac OR hemodynamic OR haemodynamic OR “blood pressure variability” OR hypertension OR “vascular function”)) AND PUBYEAR > 1999 AND PUBYEAR < 2027
Web of Science	TS=((“chronic low back pain” OR “chronic back pain” OR CLBP OR “persistent low back pain”) AND (“autonomic dysfunction” OR dysautonomia OR “autonomic nervous system” OR sympathetic OR parasympathetic OR “heart rate variability” OR HRV OR “baroreflex sensitivity”) AND (cardiovascular OR cardiac OR hemodynamic OR haemodynamic OR “blood pressure variability” OR hypertension)) Timespan: 2000–2026; Indexes: SCI-EXPANDED, SSCI, ESCI
EBSCOhost	TX (“chronic low back pain” OR “chronic back pain” OR CLBP OR “persistent low back pain”) AND TX (“autonomic dysfunction” OR dysautonomia OR “autonomic nervous system” OR sympathetic OR parasympathetic OR “heart rate variability” OR HRV) AND TX (cardiovascular OR cardiac OR hemodynamic OR haemodynamic OR “blood pressure variability” OR hypertension) Limiters: Published Date 20000101–20261231
Cochrane CENTRAL	(“chronic low back pain” OR “chronic back pain” OR CLBP) AND (“autonomic dysfunction” OR dysautonomia OR “heart rate variability” OR HRV OR “autonomic nervous system”) AND (cardiovascular OR cardiac OR hypertension OR “blood pressure variability”) Publication Years: 2000–2026
CINAHL	(MH “Low Back Pain+” OR “chronic low back pain” OR CLBP) AND (MH “Autonomic Nervous System+” OR “autonomic dysfunction” OR dysautonomia OR “heart rate variability” OR HRV) AND (MH “Cardiovascular System+” OR cardiovascular OR cardiac OR “blood pressure variability”) Limiters: Published Date 20000101–20261231
EMBASE	(‘chronic low back pain’/exp OR ‘chronic low back pain’ OR ‘chronic back pain’ OR CLBP) AND (‘autonomic dysfunction’/exp OR dysautonomia OR ‘autonomic nervous system’/exp OR ‘heart rate variability’/exp OR HRV) AND (‘cardiovascular disease’/exp OR cardiovascular OR cardiac OR ‘blood pressure variability’ OR hypertension) AND [2000-2026]/py
Google Scholar	allintitle: (“chronic low back pain” OR CLBP) AND “autonomic dysfunction” AND cardiovascular; Additional searches: “heart rate variability” chronic low back pain; “dysautonomia” chronic low back pain
OpenGrey	(“chronic low back pain” OR CLBP) AND (“autonomic dysfunction” OR “heart rate variability”) AND (cardiovascular OR cardiac); Years: 2000–2026
WorldCat	(“chronic low back pain” OR CLBP) AND (“autonomic dysfunction” OR dysautonomia OR “heart rate variability”) AND (cardiovascular OR cardiac); Year: 2000–2026
ClinicalTrials.gov	Condition: “Chronic Low Back Pain”; Other terms: “autonomic dysfunction” OR “heart rate variability” OR dysautonomia OR cardiovascular; Study Start: 2000–2026
WHO ICTRP	(“chronic low back pain”) AND (“autonomic dysfunction” OR “heart rate variability” OR dysautonomia) AND (cardiovascular OR cardiac); Recruitment Years: 2000–2026
PROSPERO	(“chronic low back pain”) AND (“autonomic dysfunction” OR “heart rate variability” OR dysautonomia); Years: 2000–2026
OSF Registries	(“chronic low back pain”) AND (“autonomic dysfunction” OR “heart rate variability” OR dysautonomia) AND cardiovascular; Year filter: 2000–2026
ISRCTN Registry	(“chronic low back pain”) AND (“autonomic dysfunction” OR “heart rate variability”) AND cardiovascular; Years: 2000–2026
